# Microbial Inoculants as Plant Biostimulants: A Review on Risk Status

**DOI:** 10.3390/life13010012

**Published:** 2022-12-21

**Authors:** Menka Kumari, Preeti Swarupa, Kavindra Kumar Kesari, Anil Kumar

**Affiliations:** 1 Department of Life Sciences, School of Natural Sciences, Central University of Jharkhand Cheri-Manatu, Kamre, Kanke, Rachi 835222, India; 2 Department of Microbiology, Patna Women’s College, Patna 800001, India; 3 Department of Bioproducts and Biosystems, School of Chemical Engineering, Aalto University, 02150 Espoo, Finland; 4 Department of Applied Physics, School of Science, Aalto University, 02150 Espoo, Finland

**Keywords:** biostimulants, biofertilizers, rhizosphere, soil microorganisms, phytostimulator

## Abstract

Modern agriculture systems are copiously dependent on agrochemicals such as chemical fertilizers and pesticides intended to increase crop production and yield. The indiscriminate use of these chemicals not only affects the growth of plants due to the accumulation of toxic compounds, but also degrades the quality and life-supporting properties of soil. There is a dire need to develop some green approach that can resolve these issues and restore soil fertility and sustainability. The use of plant biostimulants has emerged as an environmentally friendly and acceptable method to increase crop productivity. Biostimulants contain biological substances which may be capable of increasing or stimulating plant growth in an eco-friendly manner. They are mostly biofertilizers that provide nutrients and protect plants from environmental stresses such as drought and salinity. In contrast to the protection of crop products, biostimulants not only act on the plant’s vigor but also do not respond to direct actions against pests or diseases. Plant biostimulants improve nutrient mobilization and uptake, tolerance to stress, and thus crop quality when applied to plants directly or in the rhizospheric region. They foster plant growth and development by positively affecting the crop life-cycle starting from seed germination to plant maturity. Legalized application of biostimulants causes no hazardous effects on the environment and primarily provides nutrition to plants. It nurtures the growth of soil microorganisms, which leads to enhanced soil fertility and also improves plant metabolism. Additionally, it may positively influence the exogenous microbes and alter the equilibrium of the microfloral composition of the soil milieu. This review frequently cites the characterization of microbial plant biostimulants that belong to either a high-risk group or are closely related to human pathogens such as *Pueudomonas*, *Klebsiella*, *Enterobacter*, *Acinetobacter*, etc. These related pathogens cause ailments including septicemia, gastroenteritis, wound infections, inflammation in the respiratory system, meningitis, etc., of varied severity under different conditions of health status such as immunocompromized and comorbidity. Thus it may attract the related concern to review the risk status of biostimulants for their legalized applications in agriculture. This study mainly emphasizes microbial plant biostimulants and their safe application concerns.

## 1. Introduction

Plant biostimulants, as the name implies, are substances or microorganisms, which stimulate plant growth. Horticulturists coined the word biostimulant as a substance promoting plant growth that does not belong to the group of nutrients, soil improvers, or pesticides. It includes a diverse collection of compounds, substances, and microorganisms that are applied to plants to improve the crop yield, quality, and tolerance to biotic and abiotic stress [1]. According to the European Biostimulants Industry Council (EBIC) [2] “Plant biostimulants contain substance(s) and/or microorganisms whose function, when applied to plants or the rhizosphere, is to stimulate natural processes to enhance/benefit nutrient uptake, nutrient efficiency, tolerance to abiotic stress, and crop quality. Biostimulants have no direct action against pests, and therefore do not fall within the regulatory framework of pesticides” [2]. Other terminologies can also be used to define biostimulants such as biogenic stimulators [3], organic biostimulants [4], plant strengtheners [5], phytostimulators [6], and agricultural biostimulants [7]. It has been reported that the global market for biostimulants reached $2241 million by 2018 and Europe was the largest market for biostimulants in 2012 [8]. Biostimulants were later elaborated by EBIC as “Biostimulants foster plant growth and development throughout the crop life cycle from seed germination to plant maturity in several demonstrated ways, including but not limited to: improving the efficiency of the plant’s metabolism to induce yield increases and enhanced crop quality; increasing plant tolerance to and recovery from abiotic stresses; facilitating nutrient assimilation, translocation, and use; enhancing quality attributes of produce, including sugar content, color, fruit seeding, etc.; rendering water use more efficiently; enhancing certain physicochemical properties of the soil and nurturing the development of complementary soil micro-organisms” [9]. Thus, biostimulants are organic-based plant growth promoters and regulators. They provide nutrients to plants and enhance crop productivity in an eco-friendly manner. The word ‘biostimulant’ was first defined by Kauffman et al. [10] as materials, other than fertilizers that promote plant growth when applied in low quantities. They are mostly organic products and comprise amino acids, plant hormones, polysaccharides, and humic substances and are easily available for uptake by plants. They may not only deliver nutrients to the plant but also stimulates plant metabolism and alleviate biotic and abiotic stresses [11]. Parrado et al. [12] reported various mechanisms of biostimulant action through which they enhance crop yields, such as stimulation of soil microbial activity, intensification of vital soil-enzyme activities, or phytohormone production. In November 2012, the first world congress was held on the topic of the use of biostimulants in agriculture in Strasburg, France, which was participated in by 30 countries. The main intention of this congress was to bring together people working on the features of biostimulants in academia, industry, and regulatory agencies. Therefore, the uses of these biological substances became commercialized [8].

In this review, we are mainly emphasizing plant biostimulants and their role in agriculture. Besides these, we also discuss microbial inoculants as plant biostimulants, associated risk, and their biosafety regulations when applied in the agricultural field to promote plant growth.

## 2. History of Classification of Biostimulants

Classification of biostimulants is widely documented by many scientists, stakeholders, and regulators [8,13,14]. In 2012, du Jardin classified biostimulants into eight classes which were based on bibliographic analysis of plant biostimulants and microbial inoculants, and they were not included in these categories [13]. Calvo et al. [8] reviewed five different categories of biostimulants based on a critical review of selected scientific publications related to biostimulants. Furthermore, based on practical and theoretical knowledge of agricultural and horticultural biostimulant products used, du Jardin categorized them into seven categories containing substances as well as microbes [1]. According to the Agriculture and Horticulture Development Board (2017), they were further classified into two main groups, non-microbial and microbial [15]. Recently, Pascale et al. [16] classified plant biostimulants based on enhancing plant nutrition into five categories including microorganisms. The chronological order of classification of plant biostimulants and the basis of their categorization is summarized in Table 1.

## 3. Properties of Plant Biostimulants

A new category of agricultural chemicals derived from biological sources and applied as foliar applications or as seed treatments to improve the productivity of crops and overall growth and yield is known as plant biostimulants [17]. They mostly act as biofertilizers in agriculture and horticulture. There are various properties reported by many scientists of plant biostimulants which are mentioned below:

- Improving plant metabolism which induces crop yield and increases the quality of crops [18].
- Plant biostimulants protect plants against environmental stresses such as water deficiency, exposure to sub-optimal growth temperatures, and soil salinization [1].
- They are also known to promote plant growth through better nutrient uptake.
- Increasing soil enzymatic as well as microbial activities [16].
- Alteration of the architecture of roots along with enhancement of solubility and mobility of micronutrients [19,20,21].
- Enhancing fertility of the soil, predominantly by nurturing the development of complementary soil microbes [18].

## 4. Plant Biostimulants and Their Mechanism

### 4.1. Humic Substances (HS)

Humic substances (HS) are diverse organic molecules that are formed during microbial and chemical degradation of organic matter in soils and are found most abundantly in nature [21,22,23]. They also contain a total of 60% organic molecules in the world’s soils [23,24]. HS was earlier called “gelbstoff”, a yellow-colored organic compound generally found in marine water, freshwater, and soil, and made of linked polymers of amino acids, fatty acids, and carbohydrates which are resistant to further degradation by microbes [24,25]. But today, it is believed that HS is composed of small organic molecules linked together by hydrogen bonds and hydrophobic interactions [23,26,27].

Aiken et al. [28] defined HS as “a general category of naturally occurring, biogenic, heterogeneous organic substances that can generally be characterized as being yellow to black in color, of high molecular weight (MW), and refractory”. Humic substances obtained from freshwater and terrestrial ecosystems contain lignin, but it is absent in marine ecosystems [25]. Many scientists have reported that humic substances play an imperative role in the function of soil and plants [29]; for instance, by exchange of carbon and oxygen between the soil and atmosphere, and regulating the availability of nutrients [8], they enhance the physical and chemical properties of soil [16] and transform toxic materials and transport them [29,30]. In addition to this, humic substances also affect the chemical and functional properties of rhizospheric microorganisms [31]. One important feature of humic substances is the formation of complexes, although the solubility is a function of pH and the molar ratio of the complex, with micronutrients (iron) avoiding leaching, and in turn, making them available in the soil for plant nutrition [32,33]. 

Based on their solubility and molecular weight, humic substances can be divided into three groups: humic acids; humins; and fulvic acids. Humic acid is a relatively high-molecular-weight compound and is soluble in alkaline media. It can be easily extracted from soil by treating with dilute alkali and gets precipitated in acidic media, whereas fulvic acid is a low-molecular-weight compound. It is soluble in acidic and alkaline media and cannot be easily extracted [29,34]. Humin is a humic-containing substance instead of a humic substance because it is made up of humic and non-humic materials [35,36]. HS is known to increase the fertility of soil and also alleviate heavy-metal stress. Heavy metals can bind with the carboxyl and phenolic groups (binding site for heavy metal) of the humic substance, resulting in heavy-metal-deficient areas and plants which are unable to take up these metals [37]. Lead toxicity can be minimized with the application of humic and fulvic acid, which reduces the transfer and contamination of Pb^2+^ in the food chain [38,39]. Thus, humic substances have multiple roles and can be applied to stimulate the physical, chemical, and biological activity of soil and plants.

### 4.2. Protein Hydrolysates and Amino Acids

Stimulation of plant growth and tolerance to abiotic and biotic stress can also be reported using a variety of protein-based products which are different from nitrogen sources. These protein-based products can be categorized into two main groups—protein hydrolysates and amino acids. A combination of peptides and amino acids of either plant or animal origin that are manufactured from partial hydrolysis of a protein source are known as protein hydrolysates [8,16]. Some specific amino acids also function as plant stimulators such as glutamine, glycine betaine, proline, and glutamate. Protein hydrolysates are commercially available in different formulations in the form of powder, granules, and liquid and they may be applied to plants near the root system or as foliar sprays [40]. Various processes are involved in the preparation of protein hydrolysates, e.g., chemical, enzymatic, or thermal hydrolysis of plant residues (carob-germ protein, alfalfa residues, algal proteins, and wheat-condensed distiller solubles) and animal residues (connective or epithelial tissues, collagen, and elastin of animals) [8]. Protein hydrolysates are commercially available in the market of various countries with diverse names such as Aminoplant or Siapton (Italy), Macro Sorb foliar (Spain), and ILSATOP (Italy). The concentration of free amino acids and protein/peptides generally present is 2–18% and 1–85%, respectively, in protein hydrolysates preparation. Free amino acids such as arginine, alanine, valine, leucine, glutamate, proline, and alanine are the major components usually present in protein hydrolysates. In addition to protein/peptide and free amino acids of hydrolysates, some non-protein components also influence plant-growth stimulation. For instance, carob-germ extract, a plant-based product comprising carbohydrates, fats, macronutrients, and micronutrients and also containing phytohormones [8]. Another group of protein-based products is individual amino acids which include non-protein amino acids that are found extensively in a few plant species, such as glycine betaine, glutamate, histidine, and proline, render anti-stress properties [8,41]. There are many stimulatory effects of protein hydrolysates on plants such as improvement of soil respiration, increase in biomass, and activity of microorganisms because plants and microorganisms can easily utilize these amino acids and peptides as a source of nitrogen and carbon [42]. They also provide macronutrients (Ca, Mg and K) and micronutrients (Fe, Mn, Zn, and Cu) to the plants because they can chelate these metal nutrients present in soil and make them available to plant roots [1,40]. Some industries use this strategy for making biofertilizers having high nutrient efficacy. Moreover, protein hydrolysates are also known to induce the defense mechanism of plants and also increase tolerance to a range of abiotic stresses such as drought, salinity, oxidative conditions, and temperature [43,44,45,46,47]. Several higher plants that include soybean, alfalfa, rice, barley, and maize can tolerate a wide variety of abiotic stress due to the exogenous supply of these compounds [44,48,49]. Corte et al. [50], in their study, found that there was the absence of any kind of genotoxic effects shown by animal-based protein hydrolysates on soil microflora and fauna, yeast, and plant bioassay systems [49,50]. 

### 4.3. Seaweed Extracts and Botanicals

Seaweeds are also known as large marine algae including multicellular, macroscopic, and benthic organisms that inhabit the world’s oceans and provide shelter and food for oceanic animals and also offer a valuable product as single cell protein for mankind [51]. In Asian countries, fresh seaweed is mainly used for food along with traditional remedies [52]. It contains a variety of constituents, i.e., polysaccharides, proteins, polyunsaturated fatty acids (PUFA), polyphenols, pigments, and plant growth hormones [53]. In coastal regions, seaweed has been used as a fertilizer to enhance the growth of plants [54].

These liquid extracts are commercially available for horticulture and agriculture [54,55]. The extracts act by adding chelators and phytohormones and by improving soil structure and aeration [56]. Seaweed extracts are commercially made from brown algae such as *Ralfsia*, *Ascophyllum nodosum*, *Padina*, *Turbinaria*, *Sargassum*, *Laminaria*, *Fucus* spp., and others [57,58,59,60]. The seaweed extract is formulated in liquid or dried form and can be blended with micronutrients and fertilizers for field application [8]. The biostimulation effects of seaweed extracts include increasing plant growth, fruit and flower production, and crop yield, helping to develop resistance against abiotic and biotic stresses, enhancing shelf life after harvest, and increasing chlorophyll levels [8,60].

Botanicals are substances that are extracted from plants and are used in cosmetic products and pharmaceuticals, food ingredients, and plant protection products [61]. The biostimulatory effects of botanicals, except seaweed extracts, are not well understood and need to be explored. In ecosystems, plant-active compounds known as allelochemicals, which are known to mediate plant interactions, receive more attention regarding sustainable crop management [1]. Recent studies exploring biostimulatory effects of higher plant botanicals on white hat cabbage and radish have led to valuable additions for the vegetable plant under study [62,63]. Further study is required to establish the biostimulatory effects of these botanicals.

### 4.4. Chitin and Chitosan Derivatives

After cellulose, chitin is the second most copious biodegradable polysaccharide in nature and is composed of N- acetyl-d-glucosamine groups linked by β (1–4) glycosidic bonds through the activity of chitin synthases to form a linear chain [64]. It is mostly obtained from the exoskeleton of shrimp, insects, and crabs along with the cell walls of fungi and algae [65]. Chitin is mostly insoluble in water, ia a high-molecular-weight biopolymer, and has a porous structure favoring high water absorption. Chitosan is a derivative of chitin and is produced after the deacetylation of chitin which influences its chemical and biological properties and is also responsible for antimicrobial activity due to the protonation of its amino groups in solution [66]. Another derivative of chitin is oligochitosan (chitooligosaccharides), formed during the chemical and enzymatic hydrolysis of chitin and composed of mainly 3–10 saccharide residues of N- acetylglucosamine or glucosamine [67]. Biostimulatory effects of chitin and their derivatives have been reported by many researchers, and include the protection of plants from pests and diseases, enhancing the antagonistic action of microorganisms, improving the beneficial plant-microbe interactions, and regulating plant growth and development [65,68]. Shahrajabian et al. [69] reported various beneficial effects of chitin and its derivatives on vegetable crops such as increased photosynthetic activity, tolerance to abiotic stressors (salinity, drought, temperature), expression of defensive genes, increased antioxidant-enzyme activity, activation of plant innate immunity, induction of secondary metabolite synthesis, etc.

### 4.5. Antitranspirants

Chemical compounds which favor a reduction in the rate of transpiration from plant leaves are known as antitranspirants and alleviate drought stress by reducing the size and number of stomata [70]. Many chemicals reported as antitranspirants, such as chitosan, kaolin, calcium carbonate, salicylic acid, etc., are eco-friendly and increase the water-holding capacity of soil as well as reduce the rate of transpiration. Thus, the use of antitranspirants in plants increase crop yield in water- and high-temperature-stress conditions [71].

### 4.6. Microbial Inoculants

The agriculture system is heavily dependent on chemical inputs such as pesticides, herbicides, and fertilizers to enhance yield [72,73]. Due to the thrilling use of these chemicals, the quality of soil and the health of plants are being deteriorated, ultimately affecting human health. Therefore, there is a need to develop a sustainable approach to minimize the harmful effects of these chemicals and promote plant growth, and also improve soil quality without disturbing the natural ecosystem. The use of microbial inoculants is an alternative to these chemical inputs, which can act as biofertilizers, bioherbicides, biopesticides, and biocontrol agents [73]. Microbes are also one of the important categories of biostimulants for plants.

The formulations of beneficial microorganisms, which play an affirmative role in the soil biome in an eco-friendly manner, are called microbial inoculants [74]. Natural soil contains a variety of agriculturally important microorganisms that have a beneficial effect on soil and plants by providing nutrients and also protecting the plant from pests and diseases. Generally, there are two groups of microbial inoculants (i.e., biofertilizers and biopesticides), but those that function as biofertilizers are grouped under biostimulants [8,75]. They are also known as bioinoculants, which contain living organisms and promote plant growth through a variety of mechanisms, such as, increasing root growth and biomass, suppling nutrients, and also enhancing the capacity of nutrient uptake when applied to seeds, plants, or soil [76]. Microorganisms acting as biostimulants mainly belong to beneficial fungi groups including arbuscular mycorrhizal fungi and free-living bacteria [76,77,78]. Kloepper et al. [79] reported that plant growth-promoting bacteria (PGPB) and plant growth-promoting rhizobacteria (PGPR) are free-living bacteria, isolated from the rhizosphere of plants that can act as biofertilizers and stimulate plant growth. Many factors are responsible for the development of microbial inoculants as biofertilizers, such as the variety of plants [80,81], compatibility with chemical fertilizers, types of soil, and environmental conditions [8]. The activity of microbial inoculants is mostly influenced by root exudates (extracellular secretions by plants) and also serves as a substrate for the formation of biologically active substances [82]. Based on their biostimulatory effect, microbial inoculants (bioinoculants) can be divided into two groups, which are discussed below:

#### 4.6.1. Plant Growth Promoting Bacteria

Plant growth-promoting bacteria (PGPB) are beneficial, free-living, rhizoplanic, rhizospheric, and phylospheric bacteria and play a dynamic role in plant growth [83]. They belong to diverse genera such as *Bacillus* sp., *Pseudomonas* sp., *Azotobacter* sp., *Enterobacter* sp., *Azospirillum* sp., etc. Bashan and de Bashan suggested various positive effects of plant growth that include improved plant nutrition, abiotic and biotic stress [84,85] tolerance, increased growth and yield [85,86,87], and also control of plant pathogens. They are either applied either directly to the seed and plant or mixed with a carrier material such as compost, peat, sawdust, vermiculite, or compost, which provides a suitable environment for their growth [88,89]. A lot of studies have been conducted by many researchers to demonstrate the role of PGPB on plants. They promote plant growth by various mechanisms and provide nutrients to plants. These mechanisms include: (i) biological nitrogen fixation [90,91]; (ii) solubilization of inorganic P [92]; (iii) production of iron chelating compounds (iv); and phytohormones production, which is discussed in detail below:

##### Biological Nitrogen Fixation

Nitrogen is an essential component of all living organisms including plants. It is an important constituent of amino acids, nucleic acids, proteins, and energy currency (ATP, GTP, ADP), etc. Therefore, it is known as a building block of cells. Although N_2_ is present in about 78% of the atmosphere, it is unavailable for plants and animals due to its complex structure, i.e., the triple bond between two nitrogen atoms [93]. PGPB can convert this gaseous form of nitrogen into a usable form, i.e., ammonia, by the use of an enzyme system, nitrogenase, and make it available to plants. Microorganisms that fix nitrogen belong to diverse genera such as *Azotobacter* spp. [94], *Bacillus polymyxa* [95], *Gluconoacebacter diazotrophicus* [96], and *Burkholderia* spp. [97]. There is also a report that inoculation of mixed inoculants of *Gluconacetobacter diazotrophicus, Burkholderia tropica, Azospirillum amazonense, Herbaspirillum rubrisubalbicans,* and *Herbaspirillum seropedicae* has been very effective in promoting N fixation in sugar cane [98]. *Azospirillum* is the most studied nitrogen fixer among these bioinoculants as reported by Calvo et al. [8]. It has been reported that there is a major increase in nitrogen content in plants when some plant species are inoculated with *Azospirillum* strains. For instance, *A. lipoferum* and *A. brasilense* show 7–12%, and *A. diazotrophicus* gives a 60–80% increase in wheat and sugarcane, respectively.

##### Solubilisation of Phosphate

Phosphorus is also a chief nutrient for plants next to nitrogen. In agricultural soils, the total concentration of phosphorus generally varies between 400 and 1200 mg/kg^−1^ but only 1 mg/kg^−1^ is available in the forms of dihydrogen phosphate (H_2_O_4_P^−^) and hydrogen phosphate (HO_4_P^−2^) [14]. Soil contains P in inorganic and organic forms which are insoluble [14]. The inorganic form of P contains about 20–50% of total P [99] and is generally available in the form of PO_4−_ ions, which are sparingly soluble due to the adsorption of positively charged ingredients of soil and also are precipitated with some metals such as Al, Fe and Ca [99]. Insoluble organic P is also available in the form of inositol phosphates, phosphate esters, and uncharacterized large organic molecules and contains 50–80% of total P [99]. In agriculture systems, the low availability of P in soil is a significant problem [8]. PGPB can increase the nutrition of plants through the process of solubilization of P [14]. To solubilize insoluble inorganic and organic P, bacteria use several approaches. There are two mechanisms for the solubilization of P by bacteria that are predominant, i.e., through the production of organic acids or through the production of phosphatase and phytase enzymes [100,101]. Due to organic acid production, insoluble inorganic P gets transformed into soluble form due to the presence of carboxyl and hydroxyl groups that chelate the cations bound to phosphates [102] and transform them into soluble forms. The pH of immediate soil is also decreased by the production of organic acids and the solubility of P is improved by releasing phosphate ions [103]. PGPB are also known to solubilize organic P by the production of phosphatase and phytase enzymes and converting them into soluble forms [104] which plants can easily uptake from the soil. 

The predominant rhizospheric bacteria and others that have the ability to solubilize P belong to the genera *Burkholderia*, *Pseudomonas*, *Bacillus*, *Rhizobium*, *Agrobacterium*, *Achromobacter*, *Streptomyces*, *Micrococcus*, *Erwinia*, etc. Most phosphate-solubilizing bacteria that show good results under laboratory conditions, may not work well in soil conditions [99]. Therefore, rigorous field studies are ongoing to successfully characterize field-compatible phosphate-solubilizing potent bacteria.

##### Production of an Iron Chelating Compound

In the biosphere, iron is the fourth most abundant micronutrient. In aerobic conditions, iron is mostly found in ferric ions or Fe^+3^ which is insoluble and not easily accessible to plants and microorganisms [105]. In calcareous soil, Fe is not available for plants due to alkaline conditions making it less soluble [106]. Microorganisms, especially PGPB have a mechanism for producing low-molecular-weight iron-chelating compounds known as siderophore [93]. These compounds help in transporting iron into the bacterial cells and also make it available to plants. Siderophores also act as a biocontrol agent as they can create iron-deficient areas near the plant roots by inhibiting plant pathogens [105]. There is a significant increase in Fe uptake in some plants such as sunflower and maize in nonsterile calcareous soils as compared to sterile soil. This occurs due to the action of soil microorganisms that help in the uptake of Fe to plants [106]. It has been shown by Sharma et al. [107] that there is a significant increase in iron content in rice when inoculated with the strain of *Pseudomonas* because of the production of siderophores and also enhanced nutritive value of rice grains due to the increased levels of iron. Thus, siderophore production is an important trait of PGPB and enhances the iron uptake of plants.

##### Phytohormone Production

Microbial inoculants such as PGPB are also known to produce a number of plant hormones or plant-growth regulators that alter the architecture of roots and the growth of plants [108,109,110]. These plant hormones are gibberellins, auxins, ethylene, cytokinins, and abscisic acid [111]. A number of physiological processes can be regulated by these hormones, including root elongation, formation of root hairs, and root initiation [8]. Indole-3-acetic acid (IAA) has been widely reported as a natural auxin produced by microbial inoculants [112]. Many plant functions are influenced by IAA such as root initiation, differentiation of vascular tissue, expression of many plant genes, and mediation of tropic responses [8]. Cytokinins also play an important role in plants, including delaying leaf senescence and promoting mitotic cell division in roots and shoots [16]. Flower and fruit production, seed germination, and dormancy of vegetative organs are affected by gibberellin hormones [113]. Furthermore, abscisic acid is mainly involved in responses to environmental stresses such as high salinity, and drought along with plant development [114]. In addition, ethylene is well known as a ripening hormone, but there are other roles that have been reported such as cell expansion, flower and leaf senescence, and seed germination. Ethylene is also known as a stress hormone because it is produced under abiotic as well as biotic stress [115]. An inhibitory effect on root growth has also been reported due to the production of high concentrations of ethylene, which ultimately reduces plant growth. To overcome this problem, PGPB are also known to produce a vital enzyme, L-aminocyclopropane—1-carboxylate deaminase (ACC deaminase), which catalyzes the formation of the intermediate precursor of ethylene, ACC (1-amino cyclopropane-1-carboxylic acid), into α ketobutyrate and ammonia and regulates the biosynthesis of ethylene [8]. Moreover, PGPB also produced some low-molecular-weight volatile organic compounds (VOCs) such as ketones, alcohols, hydrocarbons, and aldehydes, which have generally high vapor pressure and enter into the atmosphere [116]. These compounds are collectively termed microbial volatile organic compounds (mVOCs) [117]. Initially, Fernando et al. [118] and Vespermann et al. [119] reported some biocontrol activity of these VOCs of some rhizospheric microorganisms, but later promotion of plant growth also reported the role of VOCs, for instance, the growth promotion in *Arabidopsis thaliana* by VOCs of PGPB strains containing acetoin and 2,3 butanediol [120]. PGPB also affects the morphology of roots and provides nutrition to plants. 

#### 4.6.2. Arbuscular Mycorrhizal Fungi (AMF)

**Figure 1:**
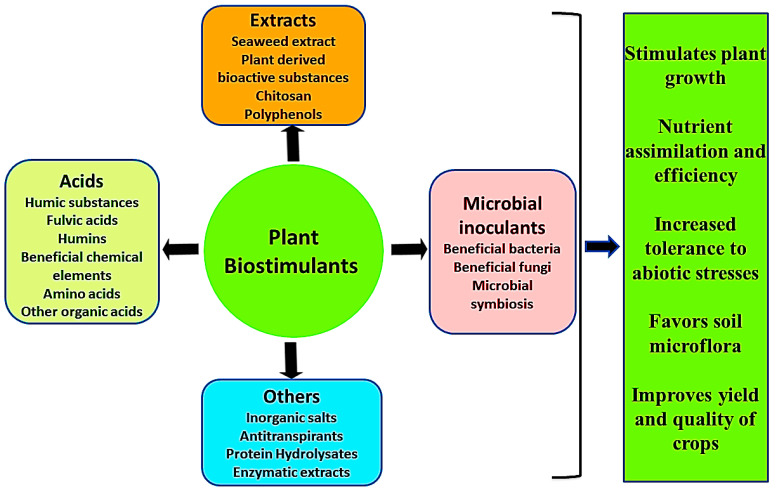
Schematic representation of the different types of plant biostimulants and their beneficial mechanisms in plants [1,8].

Fungi are also found in soil and are associated with plant roots in the following two ways: through mutualistic symbiosis and parasitism. In mutualistic symbiosis, both organisms live together and establish beneficial relationships, whereas, in parasitism association, one partner benefits and the other is harmed [121]. A beneficial and heterogeneous group of fungi that establishes symbiotic relationships with more than 90% of plant species is known as mycorrhizal fungi [1]. Mycorrhizal fungi can be categorized into different groups, but arbuscular mycorrhizal fungi are a prevalent type of endomycorrhiza and are commonly associated with horticultural and crop plants. Early in the history of land plants [122], arbuscular mycorrhizal fungi (phylum Glomeromycota) appeared first and were associated with diverse plant taxa [123]. A special branched structure formed during the penetration of fungal hyphae of Glomeromycota species in root cortical cells of plants is called arbuscules [121,124]. Today, there is great interest in the use of these mycorrhizal fungi in sustainable agriculture, which have been established to provide enhanced nutrients (macro and micronutrients) and water uptake and also help plants survive biotic and abiotic stress [125,126,127,128,129,130]. There is a recent report that not only is there interconnection between fungi and plants established by the hyphal network, but also connecting individual species of plants within a community and helping in signaling among interplant species [131,132]. AMF plays an important role in stimulating plant growth through several mechanisms [133]: (i) enhancing the uptake of water; AMF increases the surface area of the root through which plants can easily take up water; (ii) availability of nutrients, especially phosphorus, under nutrient-deficiency condition; (iv) modifications of root architecture; (v) changes in enzymatic and physiological activities, especially for plants that are involved in antioxidative responses; and (vi) induction of ABA plant hormones, which are mainly involved in stress conditions [134]. Auge, Brundertt, and Begum [125,135,136] reported some ameliorating effects of drought due to mycorrhizal symbiosis in some plant species, including wheat, onion, soybean, lettuce, and corn. This occurs due to increased root growth resulting in enhanced tolerance to drought. It also maintains high water efficiency and increased growth when plants are colonized by AMF [8]. Furthermore, the water potential of plants may also be affected by the changes in the structure of soil by the production of a soil-binding material such as glomalin, a glue-like substance that is insoluble in nature by the hyphae of AMF [125]. Protection of plant roots from the toxicity of heavy metals by the use of AMF has also been reported by Leyval et al. [137]. There are also some reports available and reporting that drought tolerance of plants is augmented by the application of co-inoculation with AMF and PGPR. For example, improved plant growth, stomatal conductance, the efficiency of water use, as well as increased photosynthetic rate, being reported in lettuce plants when co-inoculated with AMF *Glomus mosseae* and *G. intraradices* and *Bacillus* spp (PGPR). A better result was obtained in co-inoculation with AMF and *Bacillus* spp. as compared to individual organisms. This occurred due to PGPR, i.e., *Bacilus* spp., enhancing the growth and colonization of AMF [138]. But there are some limitations on the use of AMFs as biostimulants, which may result from their biotrophic character; they have difficulty for propagation on a large scale, and researchers have been unsuccessful in understanding the determinants of host specificities and other population dynamics of mycorrhizal fungi in agricultural ecosystems [139]. Some fungi which are distinct from mycorrhizal species are also reported such as *Trichoderma* spp and *Sebacinales*, which are able to colonize roots and provide nutrients to their hosts, but the mechanisms are not well studied [121]. However, these fungi can be used as bioinoculants to improve the nutritional status of plants. *Trichoderma* spp. is well known for its biocontrol and biopesticidal activities, but Colla et al. [40] and Shoresh et al. [140] also reported some stimulatory effects on plants such as enhanced efficiency of nutrients, morphogenesis, and organ growth along with increased tolerance to abiotic stress. These fungal endophytes may be considered biostimulants as well as biopesticides based on these effects on plants as reported by researchers [40,140]. Therefore, microbial inoculants including beneficial bacteria as well as fungi are a promising tool in sustainable agriculture. They not only enhance plant nutrition but also assist plants in tolerating a number of environmental stresses. They improve our agriculture system without any deleterious effects. The overall mechanisms of action of different plant biostimulants in the plant are represented below (Figure 1): 

## 5. Risk Status of Microbial Inoculants (Plant Growth Promoting Bacteria)

PGPB, which are considered potent candidates for plant growth, should be safe for mammals. Some of the microbial inoculants commonly used as biostimulants, and their risk groups are listed in Table 2. Despite their array of beneficial effects on plants (Table 2), they may pose a risk to other living organisms, especially human beings. Although most PGPB do not have a negative effect, some genera are involved in causing infections in animals and humans. Bacteria belonging to the genera *Serratia, Acinetobacter, Bacillius cereus*, *Stenotrophomona, Enterobacter, Herbaspirillum, Ochrobactrum,* and *Pseudomonas* are not only powerful candidates for plant-growth promotion but may also cause disease in humans [141,142]. *Pseudomonas,* besides being a potential candidate as PGPR, is also responsible for many types of opportunistic infections in humans who are aged, immunocompromised, or suffering from conditions such as cancer, severe burns, or cystic fibrosis. Some common pathogenic species of *Pseudomonas* are *P. cepacia, P. aeruginosa, P. putida, P. fluorescens,* etc [143]. Although *Bacillus* sp. is commonly known for its wide variety of applicability in agriculture, industry, and the pharmaceutical sector, it still is associated with many types of illness in humans and animals. It can cause disease in immunocompromised as well as in healthy individuals. Some species may cause minor infections, but some species may be associated with severe or lethal infections. *B pumilus, B licheniformis, B coagulans,* and *B thuringiensis* are examples of *Bacillus* species that are associated with various infections [144]. *Aeromonas* sp. *is* used as PGPR but also causes diseases in immunocompetent and immunocompromised people such as septicemia, gastroenteritis, and wound infections [145]. Another potent PGPR, *Comamonas* spp., is also associated with many life-threatening illnesses such as endocarditis, and septicemia in immunocompetent individuals [146]. *Streptomyes* sp. can cause changes in tissue structure in humans leading to diseases such as cancers, mycetomas, and actinomycetomas [147]. In spite of such immense positive impact of *Trichoderma* sp. on plant health, it is now emerging as a human pathogen causing diseases such as peritonitis, subcutaneous infections, and hematologic disorders [148]. Although *Enterobacter* sp. has a variety of uses as a plant growth stimulator, is also known to cause nosocomial infections and is involved in an array of ailments such as skin infections, inflammation in the respiratory system, and meningitis in neonates, immunocompromised individuals and hospitalized patients [149]. But there are no national or international rules or regulations to assess the risk associated with the commercial use of these plant-beneficial microbes [150]. Even commercial biofertilizers such as Biosubtilin, Nitrofix, and Bioderma. (Table 2) do not mention risks associated with the respective inoculants in their packets.

Risk groups and biosafety levels (BSL) are two terminologies used to describe and categorize microbes as per the level of hazards they can cause [93]. According to the World Health Organization (WHO 2015), microorganisms that are categorized under various risk groups (RG) are based on certain criteria such as their pathogenicity and virulence, host range, mode of transmission, availability of vaccines for effective prevention, availability of medications, etc. Thus, the classification, e.g., from RG-1 to RG-4 articulate the level of hazard a particular microorganism causes. RG-1 refers to a group of microbes that do not cause or are not associated with any type of illness in healthy animals (including humans). Microbes under RG-2 group are associated with a disease that is generally mild and there are medications readily available to treat the disease. RG-3 microbes are concomitantly associated with a serious and lethal disease that may or may not be treatable. Microbes belonging to RG-4 category have the ability to cause fatal and deadly diseases for which treatment is rarely found. Biosafety level (BSL), e.g., from BSL-1 to BSL-4, is a precautionary procedure and protocol used to avoid or prevent risks associated with these risk groups while handling them. Organisms belonging to BSL-1 are nonpathogenic in nature and can be easily handled in the laboratory through general laboratory guidelines. Microorganisms under BSL-2 have the ability to cause disease in a healthy individual, but there are ample medications and vaccines available to easily cure such diseases. Proper laboratory guidelines and special training are required to handle BSL 2 organisms. Specialized safety measures and containment facilities are required to handle microbes that come under the BSL-3 group because such microbes can cause fatal infections but do have effective remedies and anticipatory treatments available. BSL-4 encompasses high-risk-associated organisms, which have aerosol-transmission ability and for which effective treatment is not available. Laboratory personnel handling such organisms must have special training and should know the primary and secondary containment of BSL-4 organisms. The literature suggests that many bacteria isolated from the rhizosphere, soil, and water, besides having PGP activity, are also involved in causing diseases in immunocompromised and healthy individuals [151,152,153]. Hence there is an immense need to develop a systemic and polyphasic approach through which we can check the disease-causing ability of microbes isolated from an environmental niche in addition to checking their PGP activity and bioinoculant development [154]. A study by Vílchez et al. stabilized a polyphasic protocol called EHSI (environmental and human safety index) to check the biosafety level of plant-growth-promoting bacteria. EHSI articulates the overall effect of PGPB on soil microflora, beneficial macroflora and fauna, and animal and human health. In this study, according to EHSI, both being potent PGPB, *Pseudomonas putida* KT2440, is relatively safe as compared to *Burkholderia cepacia* CC-Al74. In another study by Kim et al. [155], it was suggested to assess or check for the presence of genes involved in the virulence or pathogenicity of novel bacteria isolates to determine their safety level concerning humans and plants. Keswani et al. [150] suggested that whole-genome sequencing of a bacterial isolate is the best way to obtain a complete understanding of its phylogenetic categorization and pathogenic behavior. Hence, research organizations and institutions which are involved in isolating novel microbial isolates and bioinoculant development, after thorough polyphasic characterization, should use isolates that belong to BSL-1 and Risk group-1 for bioformulation because they will pose minimum risk to the environment and human health [150].

**Table 2:** Microbial inoculants in agriculture and horticulture systems and their indicative risk status in the risk group databases.

Microbial Inoculants in Research and Commercial Biofertilizers (Risk Group by TRBA/ATCC/ZKBS)	Commercial Status/Formulation (Brand Name and Manufacturer)	Plants	Effects on Plants
*Pseudomonas putida* [107] (RG2^G^/BSL1/RG2) [156,157,158] #BSL 1- *P. putida* (Trevisan) *migula*	Yes/Powder (Pseudomonas putida, Organoponix private Limited, Orissa [159]	Rice	Increased iron uptake
*Pseudomonas fluorescens* [160,161,162] (RG1/BSL1/- [156,158,163] #BSL-1- *P. fluorescens migula*	Yes/Powder and Liquid (PSEUDOMONAS FLUORESCENS Bacterial biocontrol agent, Manidharma Biotech Private Limited, Tamil Nadu, India) [164]	Rapeseed, sweet potato, rice	Increased plant height, biomass, grain yield
*Streptomyces strain* [165,166] (RG1/BSL 1/RG1) [156,158,167] #BSL 1- *Streptomyces azureus* Kelley et al.	No/-	Tomato and rice	Plant growth
*Azospirillum brasilense* Sp245 [115] (RG 1/BSL 1/RG1) [115,156,158,168] #BSL1- *A. brasilense*	Yes/Liquid (Sardar Liquid Biofertilizers- *Azospirillum* culture, Gujrat State Fertilizers, and Chemicals, India) species and strain not specified) [169]	Spring wheat	The increased dry weight of the shoot and leaf length
*Aeromonas* spp [170] (RG 1/BSL 2/RG2) [156,158,171] #BSL 2- *Aeromonas hydrophila* (Chester) Stanier	No/-	Rice	Increased root area
*Comamonas acidovorans* [172] (RG1^G^/BSL1/RG2) [156,158,173] #BSL1- *Comamonas* sp.	No/-	Lettuce	Plant growth promotion such as IAA production
*Bacillus subtilis* [174] (RG1/BSL1/RG1) [156,158,175] #BSL1- *B. subtilis* (Ehrenberg) Cohn	Yes/Aqueous suspension and wettable powder (Biosubtilin, Biotech International Limited, New Delhi, India) [176]	Lettuce	Increased cytokinin content in roots and shoots
*Bacillus licheniformis* [177] (RG1/BSL1/RG1) [156,158,178] #BSL1- *B. licheniformis* (Weigmann) Chester	No/-	Cucumber	Increased fresh weight, higher chlorophyll content, and enhanced cell division
*Azospirillum lipoferum* [179] (RG1/BSL1/RG1) [156,158,180] #BSL1- *A. lipoferum* (Beijerinck)	Yes/Carrier powder, soluble powder, and soluble liquid (Nitrofix, Agri Life, Andhra Pradesh, India) [181]	Maize seedlings	Increased root hair density
*Azospirillum lipoferum* [182] (RG1/BSL1/RG1) [156,158,180] #BSL1- *A. lipoferum* (Beijerinck) Tarrand et al.	Yes/Carrier powder, soluble powder, and soluble liquid (Nitrofix, Agri Life, Andhra Pradesh, India) [181]	Wheat	Increased tolerance to salinity conditions
*P. putida* [183] (RG2^G^/BSL1/RG2) [156,157,158] #BSL1- *P. putida* (Trevisan) *migula*	Yes/Powder (Pseudomonas putida, Organoponix private Limited, Orissa) [159]	White clover	Increased root and shoot biomass and water content
*B. megaterium* [183] (RG1/BSL1/RG1) [156,158,184] #BSL1- *B. megaterium* de Bary	Yes/Carrier powder, soluble powder, and soluble liquid (P Sol B^®^, Agri Life, Andhra Pradesh, India) [185]	White clover	Increased root and shoot biomass and water content
*Alternaria* sp. [186,187] (-/BSL1/RG1/2) [158,188]	No/-	Wheat	Stimulate drought tolerance
*Trichoderma* sp. [40,140,186,187] (-/ BSL1/RG1) [158,189] #BSL1- *T. harzianum* Rifai	Yes/Wettable powder and Aqueous suspension (Bioderma, Biotech International Limited, New Delhi) [190]; Ecosom^®^- TV, [191]; Ecosom^®^-TH [192] (Agri Life, Andhra Pradesh, India)	Barley	Increased drought tolerance
*Azoarcus* sp. [193] (RG1/BSL1/RG1) [156,158,194] #BSL1- *A. oleivorans*	No/-	Wheat	Enhanced plant nitrogen nutrition and root growth and alleviate the nutrient deficiency
*Azorhizobium* sp. [195] (RG1/BSL1/-) [156,196] #BSL1- *A. caulinodans* Dreyfus et al.	No/-	Wheat	Enhanced plant nitrogen nutrition and root growth and alleviate the nutrient deficiency
*Azospirillum* sp. [193] (RG1/BSL1/RG1) [156,158,168,180] #BSL1- *A. lipoferum* (Beijerinck) Tarrand et al., *A. brasilense* Tarrand et al.	Yes/Liquid (Sardar Liquid Biofertilizers- *Azospirillum* culture, Gujrat State Fertilizers, and Chemicals, India) [169]	Wheat	Enhanced plant nitrogen nutrition and root growth and alleviate the nutrient deficiency
*Bradyrhizobium* sp. (RG1/BSL1/RG1) [156,158,197,198,199] #BSL1- *Bradyrhizobium* sp.	No/-	Mungbeans	Increases growth parameters and seed yield
*Rhizobium meliloti* [200,201] (RG1/BSL1/RG1) [156,158,202] #BSL1- *Rhizobium* sp.	Yes/Aqueous suspension and wettable powder (Biobium Biofertilizers, Biotech International Limited, New Delhi), Species not specified [203]	Peanuts	Increases plant growth, quality of pods enhanced, and efficiency in the use of nitrogen
*R. leguminosarum* [204] (RG1/BSL1/RG1) [156,158,205] #BSL1- *R. leguminosarum* jordan	Yes/Aqueous suspension and wettable powder (Biobium) Species not specified [203]	Soybean	Increases growth and yield performance under drought stress
*Bacillus* spp. [206,207] (RG1/BSL 1/ RG1/2/3) [156,158,208]	Yes/Carrier powder, soluble powder, and soluble liquid (Si-Sol B TM, Agri Life, Andhra Pradesh, India) [209]	Strawberry	Increases fresh and dry weight parameters, increases yield
*Azotobacter chroococcum* [210] (RG1/BSL1/RG1) [156,158,211] #BSL1- *A. chroococcum Beijerinck*	Yes/Liquid (Reap^®^-N1, NCS Green Earth Private Limited, Maharashtra) [212]	Maize	Increased shoot and root length, leaf and root number, chlorophyll content
*Azotobacter vinelandii* [210] (RG1/BSL1/RG1) [156,158,213] #BSL1- *A. vinelandii* Lipman	Yes/Carrier-based powder (Nitrofix ^®^, Agri Life, Andhra Pradesh, India) [181]	Maize	Increased shoot and root length, leaf and root number, chlorophyll content
*Bacillus halotolerans* [204,214] (RG1/-/-) [156]	No/-	Wheat and soybean	Improved germination, growth, and yield, better draught resistance, improved nitrogen, potassium, and Zn uptake
*Enterobacter hormaechei* [204,214] (RG2/BSL2/-) [156,215] #BSL 2- *E. cloacae* (Jordan) Hormaeche and Edwards	No/-	Wheat and soybean	Improved germination, growth, and yield, better draught resistance, improved nitrogen, potassium, and Zn uptake
*Pseudomonas frederiksbergensis* RG2^G^ * [204,214] (RG1/BSL1/-) [156,216] #BSL1- *P. frederiksbergensis* Andersen et al.	No/-	Wheat and soybean	Improved germination, growth, and yield, better draught resistance, improved nitrogen, potassium, and Zn uptake

Risk group * (classification of prokaryotes into risk groups under Biological Agents Ordinance: RG 1 refers to prokaryotes that generally do not cause infectious disease in humans; RG 2 refers to those microbial groups which do not pose a significant risk to laboratory workers but may cause disease if there is exposure and for which there are therapeutic interventions available), RG—Risk group, BSL—Biosafety level as per ATCC; #—The exact name of the organisms in the original concerned database of risk group; (-) indicates that it has not been commercially formulated.

In addition to posing health risks to animals, unprecedented use of PGPR also affects other biotic communities of an ecosystem, especially soil resident flora. As it is already known that newly introduced microorganisms change the microenvironment of soil, creating their niche which can have an immense effect on the structure and composition of resident microbes [217]. The interaction of PGPR with soil flora may be negative, positive, or neutral depending upon the nature of the PGPR introduced into the soil [218]. The main concern is the introduction of antimicrobial-producing PGPR in the soil milieu [219]. A study by Walsh et al. [220] revealed that there was a reduction in the diversity of the rhizobacterial population due to the introduction of 2,4-diacetylphoroglucino (an antibiotic substance) producing bacteria in the rhizosphere. Some type of perturbance in the resident-flora population is also possible as found in the study by Albright et al. [221]. 

## 6. Safety Determination of Microbial Inoculants

Several microbes belonging to *Pseudomonas, Bacillus, Acinetobacter, Burkholderia, Staphylococcus, and Stenotrophomonas* have been used as inoculants for plant-growth promotion and biocontrol of plant pathogens; however, these also include microbes identified as opportunistic pathogens and that cause human pathogenesis [150]. It has also been reported that the invasion and colonization mechanisms involved in the pathogenesis of PGPR on plant and human tissues are similar [151,222,223]. Therefore, the safe application of PGPR to protect human health and the environment is needed, which involves collaborative efforts of different expertise, and technological advancements. Microbial inoculants need to be identified and well characterized to unveil their hidden risks to humans and the environment. Several physiological and molecular approaches are now used to check the virulence and pathogenicity of infectious microbes. These methods can also be employed to detect the pathogenicity level of PGPB. The following are some important detection methods that can be taken into consideration.

### 6.1. Morphological and Biochemical Methods

To detect the pathogenicity level of bacteria, it is necessary to identify the species of bacteria which can be done through cultural studies and fast biochemical tests. For example, growth on blood agar will indicate that the bacteria are pathogenic in nature. The use of differential and selective media will enhance the probability of isolating microbes that have a pathogenic nature. Various biochemical tests such as tests for enzyme detection of catalase, urease, deaminase, decarboxylase, deaminase, β galactosidase, hydrolase, etc., are helpful in the polyphasic characterization of bacteria. These enzymes can also be detected using chromogenic media that contains specific chromogenic substrates which are hydrolyzed and produce a particular color in the media indicating the presence of enzymatic activity in bacteria. Nowadays various biochemical kits and their detecting instruments are available commercially, which enables the rapid detection of microbes [224,225,226].

### 6.2. Antibiotic Sensitivity Method

Sensitivity to various antibiotics will indicate whether the given bacterial isolate is safe for release into the environment or not because multiple drug-resistant PGPB bacteria that somehow cause disease in humans and animals will be difficult to treat or cure such disease through prevalent antibiotics. In addition, antibiotic resistance is generally plasmid-borne and most of the plasmid can be transferred from one bacterium to another, thereby spreading the antibiotic-resistant character in the soil microbiome [227]. 

### 6.3. Protein Profiling Method

Every genus has a particular set of proteins, and protein profiling will help in identifying the bacterial genus. Even various species in one genus can be differentiated through protein profiling as they have a particular set of proteins, i.e., they contain enzymes involved in a unique biochemical pathway. In addition, it may be possible that these unique biochemical pathways enable a particular microbe to thrive in a harsh climate making them a more favorable candidate for bioinoculant production [227].

### 6.4. Molecular Level Detection Techniques

Studying at the genetic level is the most precise, rapid, and sensitive technique in today’s era to help in the proper understanding and identification of microbial species. Detection of ubiquitous and universal sequences (containing conserved and variable regions) such as 16s rDNA/18s rDNA is the most prominent and simple way to identify microbes at the species level. Techniques based on the hybridization process are used to detect genes of interest through the use of probes tagged with fluorescent dyes. For example, the fluorescence in situ hybridization (FISH) method uses universal probes to detect a particular microbe [228,229].

Amplification of genes conferring the virulence property of a particular microbe is also an effective way to check the pathogenic nature of bacteria. Quantitative polymerase chain reaction (qPCR) and reverse transcriptase real-time PCR (RT-qPCR) are employed as amplification techniques. One such example is an *invA* gene, which is a virulence gene found in *Salmonella* sp. that is detected through PCR using compatible primers [230].

**Figure 2:**
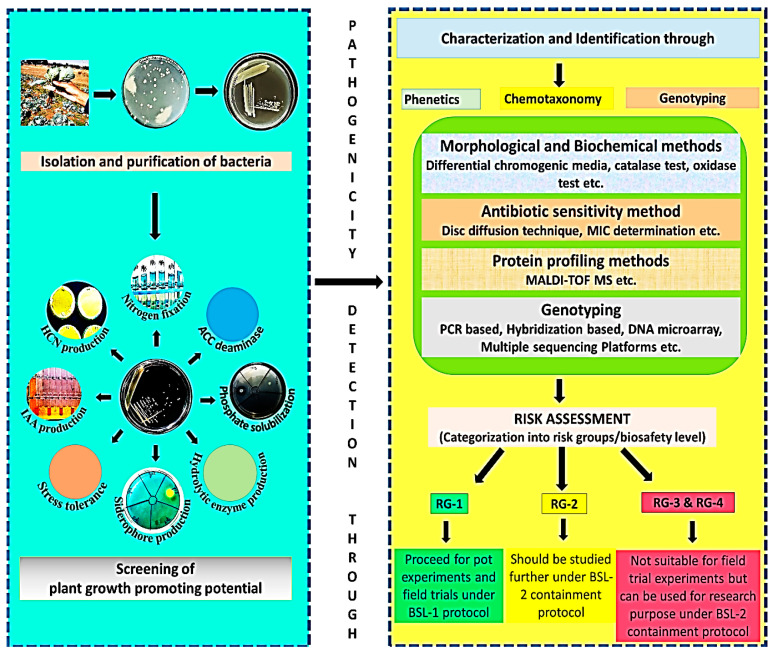
A graphical abstract of isolation, characterization, and identification of microbial inoculants and their commercialization as per their respective categorization into different risk groups (compiled from) [150,227,237,238].

Gene chip technology or DNA microarray is yet another efficient technique that can not only identify and differentiate among various species of microbes through a variety of probes and universal or consensus primers, but can also give information regarding different resistant measures adopted by a specific microbe [231]. With the advent of the Sanger method of sequencing, a first-generation sequencing technique, it is now possible to sequence the whole genome of a particular microorganism in a very rapid and efficient way. The sequence of the whole genome will not only identify the bacterium but also disclose its pathogenic nature and resistant profile. Whole-genome sequencing also helps in the rapid designing of primers [232,233]. Nowadays, NGS (next-generation sequencing) has proven to be a powerful method for the detection of virulence factors of infectious microorganisms within a few hours. In clinical microbiology, there are numerous methods available for the detection of human pathogens, which are compiled in Table 3. These technologies, in combination with the routine characterization and evaluation of potential microbial biostimulants, can be used to guide as per Figure 2 for the safe development and enrichment of microbial stimulants for use in agriculture.

**Table 3:** Comparative table of different technological approaches for the detection of pathogenic organisms.

Technological Approach	Major Targets for Pathogens Detection	Advantage/Limitations	References
**Phenotypic methods**			
(i) Morphological and biochemical methods	Metabolic potential and specific enzymes such as catalase, oxidase, phosphatase, hydrolase enzymes, etc.	Traditional low-cost, easy-to-operate, standardized methods cannot differentiate between target and non-target endogenous microorganisms, time and labor-consuming procedures, and also unable to detect viable unculturable organisms	[227]
(ii) Antibiotic-sensitivity testing	Resistant markers transmission		
**Protein profiling method (Proteomics)** MALDI-TOF MS	Specific proteins of particular bacteria to identify specific genera and species.	Qualitative and quantitative determination of proteins in most clinical laboratories. Low concentration of proteins leads to errors in the data interpretation (resistant mechanisms). Unable to differentiate taxonomically related bacteria	[227,234,235]
**Molecular methods (genomics)**			
(i) Amplification methods: Quantitative real-time polymerase chain reaction (qPCR), reverse transcriptase real-time PCR (RT-qPCR), and Loop-Mediated Isothermal Amplification (LAMP)	hybridization between the target nucleic acid and the pathogen-specific probe	More sensitive methods for the identification of pathogens at the molecular level suffer in case of low concentrations of pathogens.	[228,231,236,237]
(ii) Hybridization-based methods			
(iii) DNA microarrays (gene chip technology)	hybridization between the target nucleic acid and the pathogen-specific marker gene panels.		
(iv) Whole-genome sequencing	whole genome sequence	Identification of pathogens, profiling of resistant genes, recognition of outbreaks, and immediate design of PCR probes based on the generated genetic data in the outbreaks.	
(v) Next-generation sequencing			
**Microfluidics based methods**	It is a multidisciplinary strategy and utilizes pathogen markers	extraction and identification of pathogens from clinical/environmental samples.	[238,239]

MALDI-TOF MS- Matrix-Assisted Laser Desorption/Ionization-Time-Of-Flight mass spectrometry, MS-Mass spectrometry.

## 7. Legal Framework of Biofertilizer Implementation in Different Countries

In spite of having so many advantages over chemical pesticides, the biofertilizer industry faces too many legal obstacles to overcome before entering into commercial production. Earlier the legal regulations regarding biofertilizer use were very inadequate and weak. But in today’s era, as researchers have shown the great potential of biofertilizers, many countries amended and developed strong policies and legal regulations to increase the usage of biofertilizers [240,241].

Having an appropriate legal definition is a crucial part of making biofertilizers an appealing commercial product to the producers. In the USA and European Union (EU), there is no proper definition of biofertilizers that can define their actual characteristics. In the EU, biofertilizer comes under e EU Commission Regulation n. 889/2008 on organic production, which states that biofertilizers can only be used as plant protectants against pests and diseases. Hence, biofertilizer comes under the legal agenda of plant protection products. The same outline is followed by the US National Organic Program which categorizes biofertilizers as biological organisms that can only be used as plant protectants [242].

Compared to other countries, India has the most comprehensive and defined legal regulation and framework for biofertilizer implementation. In India, biofertilizer comes under the Essential Commodities Act of 1955, Ministry of Agriculture, and can be defined as “the product containing carrier based (solid or liquid) living microorganisms which are agriculturally useful in terms of nitrogen fixation, phosphorus solubilization or nutrient mobilization, to increase the productivity of the soil and/or crop”. Seven standard criteria have been set to formulate a biofertilizer that includes viable inoculum density, the physical form, level of contamination, pH, moisture content, the particle size of carrier-based products, and efficacy level. Four groups of microbes are mainly included under the biofertilizer category i.e., *Azotobacter, Rhizobium, Azospirillum*, mycorrizal fungi, and phosphate-solubilizing bacteria [242,243]. 

In Poland, Polish Law on Fertilizers and Fertilization 2007 includes biofertilizers under “growth stimulators” and groups them under plant conditioners. This law defines biofertilizer as “a positive impact on plant growth or other metabolic processes of plants in other ways than plant nutrients” and shall “pose no threat to [the] health of humans or animals or to the environment after their use and storage instructionss” [242].

Spain, which is one of the leading countries in organic farming, does not have a separate category and definition of biofertilizer in its legal structure. It includes microorganisms as one of the components of compost and organic amendments under Real Decreto 506/2013 [242].

China has a strict and defined legal framework for biofertilizer implementation. It has set various parameters through which it can access the quality of biofertilizer including inoculum density, water, and carbon content, outer appearance, granule size, contamination, viability, and validity. Chinese standards mostly rely on the amount of inoculum to access the quality of biofertilizer, which should range between >1.5 × 109 CFU mL^−1^ or >0.2 × 109 CFU g^−1^ and >0.5 × 109 CFU mL^−1^ or >0.1 × 109 CFU g^−1^, for solid and liquid products, respectively. Seven categories of microorganisms are included in biofertilizers, i.e., fast- and slow-growing species of rhizobia, organic and inorganic phosphate-solubilizing bacteria, nitrogen-fixing bacteria, silicon-solubilizing bacteria, and various consortia containing multiple microorganisms [244].

## 8. Conclusions

Plant biostimulants prove beneficial to plants by improving their growth. Microbial inoculants, single or consortia, naturally improve plant growth and performance without using any agrochemicals in the field. They can act as biofertilizers, soil improvers, growth regulators, stress relievers, and biocontrol agents. However, more research needs to explore and establish their biocontrol properties. Much research has been conducted to understand their properties and functions followed by their commercialization to promote eco-friendly and safe agriculture practices for the fortification of plants with nutrients. The global markets of biostimulants also need to be expanded in the near future so that farmers can easily buy these products at affordable prices. Furthermore, extensive characterization research emphasizing the safety issues of the inoculant microbes becomes inevitable to address recent reports of many inoculants belonging to either higher-risk groups or potential pathogens of human beings, such as *Pueudomonas*, *Klebsiella*, *Enterobacter*, *Acinetobacter,* etc., which may cause various kind of suffering, for example, septicemia, gastroenteritis, wound infections, inflammation in the respiratory system, meningitis, etc., of varied severity under different conditions of human-health status, such as immunocompromized and comorbidity with other diseases, etc. Advances in technologies including biochemical, immunological, proteomics, and genomics approach unraveling the characters and identification of microbes have enabled the research community to rapidly and accurately address safety concerns, such as pathogenicity, of biostimulant microbes following a suitable strategic plan before releasing the inoculant for field application.

## Figures and Tables

**Table 1:** Chronological order of classification of biostimulants.

Year	2012 [13]	2014 [8]	2015 [1]	2017 [15]	2018 [16]
**Basis of categorization**	Bibliographic analysis	A critical review of selected scientific publications	Substances and microorganisms	Type of products	Plant nutrition
**Role of microbes**	No	Yes	Yes	Yes	Yes
**Categories**	1. HS 2. Complex organic materials 3. Beneficial chemical elements 4. Inorganic salts 5. SWE 6. Chitin and chitosan derivatives 7. Antitranspirants 8. Free amino acids and other N-containing substances	1. Humic acids 2. Fulvic acids 3. Microbial inoculants 4. PHs and amino acids 5. SWE	1. Humic and fulvic acids 2. Protein hydrolysates and other N-containing compounds 3. Seaweed extracts and botanicals 4. Chitosan and other biopolymers 5. Inorganic compounds 6. Beneficial fungi 7. Beneficial bacteria	1. Non-microbial (i) SWE (ii) HS (iii) Phosphite and other inorganic salts (iv) Chitin and chitosan derivatives (v) Antitranspirants (vi) PHs and free amino acids (vii) Complex organic materials 2. Microbial (i) PGPR (ii) Non-pathogenic fungi (iii) AMF (iv) Protozoa and nematodes	1. HS 2. PHs 3. SWE 4. PGPR 5. AMF

HS—Humic substances, PHs—Protein hydrolysates, SWE—Seaweed extract, PGPR—Plant Growth Promoting Rhizobacteria, AMF—Arbuscular Mycorrhizae Fungi.

## Data Availability

Not applicable.
